# GNSS Spoofing Detection Based on Coupled Visual/Inertial/GNSS Navigation System

**DOI:** 10.3390/s21206769

**Published:** 2021-10-12

**Authors:** Nianzu Gu, Fei Xing, Zheng You

**Affiliations:** 1 Department of Precision Instrument, Tsinghua University, Beijing 100084, China; gnz16@mails.tsinghua.edu.cn (N.G.); yz-dpi@mail.tsinghua.edu.cn (Z.Y.); 2 Beijing Innovation Center for Future Chips (ICFC), Beijing 100084, China

**Keywords:** GNSS spoofing, spoofing detection, coupled visual/inertial/GNSS system, visual inertial odometry, Chi-square testing

## Abstract

Spoofing attacks are one of the severest threats for global navigation satellite systems (GNSSs). This kind of attack can damage the navigation systems of unmanned air vehicles (UAVs) and other unmanned vehicles (UVs), which are highly dependent on GNSSs. A novel method for GNSS spoofing detection based on a coupled visual/inertial/GNSS positioning algorithm is proposed in this paper. Visual inertial odometry (VIO) has high accuracy for state estimation in the short term and is a good supplement for GNSSs. Coupled VIO/GNSS navigation systems are, unfortunately, also vulnerable when the GNSS is subject to spoofing attacks. The method proposed in this article involves monitoring the deviation between the VIO and GNSS under an optimization framework. A modified Chi-square test triggers the spoofing alarm when the detection factors become abnormal. After spoofing detection, the optimal estimation algorithm is modified to prevent it being deceived by the spoofed GNSS data and to enable it to carry on positioning. The performance of the proposed spoofing detection method is evaluated through a real-world visual/inertial/GNSS dataset and a real GNSS spoofing attack experiment. The results indicate that the proposed method works well even when the deviation caused by spoofing is small, which proves the efficiency of the method.

## 1. Introduction

Global navigation satellite systems (GNSSs) are some of the most important absolute positioning infrastructures, especially for unmanned air vehicles (UAVs) and other unmanned vehicles (UVs). GNSSs can provide precise position information for long periods of time, and the cost of the navigation receiver as a user-end device is low, which greatly expands the applications of GNSSs [1].

Although the GNSS has incomparable advantage, its defects also should not be ignored. GNSS navigation relies on navigation signals transmitted by satellites from Earth orbits. The transmission power of the navigation signal is limited by the volume, mass, and payload of the satellite, and the signal attenuation after passing through the ionosphere, troposphere, and atmosphere is extremely serious. The receiver is vulnerable to interference or attack. The safety of GNSSs has attracted more and more concern in recent years. GPS spoofing is one of the most important threats affecting the positioning process in GPS receivers [2].

There are two main types of GNSS interference: suppression (jamming) interference and spoofing interference. Compared with suppression interference, spoofing interference is more concealed and harmful, and anti-spoofing interference methods are more complicated. The purpose of spoofing interference is to deceive the receiver. The spoofing signals are usually created by imitation satellite navigation signals or delayed real navigation signals. The characteristics of the spoofing signals, like phase, frequency, Doppler frequency shift, and others, can be similar to the real signals when the spoofer has been synchronized with real signals. The signal-to-noise ratio (SNR) of the spoofing signal can be set slightly higher than that of the real one, so the receiver can easily lock the spoofing signals and output incorrect positioning solutions. Most UVs and UAVs utilize GNSSs as the only absolute positioning method, so spoofing attacks can damage the whole navigation system without the user being aware. To handle this kind of threat, the application of anti-spoofing technology in modern GNSSs is acquiring more and more attention [3].

In December 2011, Iran announced the capture of a state-of-the-art US RQ-170 “Sentinel” stealth drone. According to an Iranian engineer who claimed to have participated in the capture mission [4], they deployed a Global Positioning System (GPS) spoofing attack signal to induce the drone to use the coordinates set by Iran as the coordinates of the US Afghan base when undertaking the automatic return mission. The navigation data of the GPS were modified so that the estimated position of the drone was induced to the spoofer-specified one. In June 2012, Shepard et al. [5] successfully demonstrated the spoofing of drones at the US Army’s White Sand Missile Range under the supervision of the US Department of Homeland Security. The drone was set to hover mode and spoofing signals were sent to the drone to mimic GPS signals, making the drone mistakenly think that it was moving upwards; as a result, the drone moved downwards to correct itself and finally touched ground. A research team at Austin’s Radio Navigation Laboratory led by Professor Todd Humphreys of the University of Texas has, using GPS spoofing, successfully deceived drones [6] and the White Rose yacht [7] and was invited to the US Congress discussion on drone safety.

Spoofing detection methods are generally divided into three categories according to the different detection principles. The first kind is based on detecting abnormal changes in signal characteristics, such the carrier-to-noise ratio [8], signal power [9], arrival directions [10,11], Doppler effect carrier frequency shift [12], and so on. This kind of detection technology has the advantage of not requiring other auxiliary information and only utilizing the characteristics of the signal itself. However, the spoofing attacker may have the ability to acquire the receiver’s position and the speed to formulate an advanced spoofing strategy, in which a spoofing signal with similar characteristics to the real signals is generated.

The second kind of detection method is based on the encrypted information in the signal. Previous authors [13,14,15] have discussed the application of signal encryption technology to civilian GNSS receivers. Two studies [16,17] introduced a form of spoofing detection technology using symmetrically encrypted GNSS signals, deploying correlation analysis with receivers known to be affected by spoofing interference. Another study [18] used an encrypted spreading code, in which part of the spreading code is made public and the other part of the key is distributed to the receiver. This kind of method needs to modify the signal structure or navigation message structure, which is difficult to implement in the short term.

The third kind of spoofing detection method is based on utilizing supplementary measurements from other navigation or positioning sensors of the system. One previous study [19] mentioned the use of the receiver’s clock to monitor the abnormal changes in satellite time information. Others [9,17,20] concentrate on utilizing the auxiliary measurement information obtained by the inertial measurement unit (IMU). For integrated IMU/GNSSs, a Kalman filter (KF) or extended Kalman filter (EKF) is generally used for information fusion. The spoofing detection method based on the IMU always monitors the residuals of the KF or EKF measurements. A previous study [21] introduced a method of providing auxiliary measurement information by means of a land-based Roland system. 

The purpose of a spoofing attack is to mislead the navigation system by inducing the system to estimate an incorrect positioning result. The supplementary measurement methods focus on monitoring the abnormal deviation and the credibility of positioning solutions, highlighting the most important point of the spoofing detection problem. Although the IMU method is used most extensively, it also has some disadvantages restricting its detection performance in some spoofing scenarios. Due to the serious cumulative error, the IMU only has good short-term accuracy. Over time, the positioning solution output of the IMU develops a large deviation. This restricts performance in the detection of induced spoofing signals [22], which deviate from real signals slowly. 

The IMU and cameras are two of the most widely used sensors for UVs and UAVs. Spoofing detection methods based on visual measurements have also been introduced. Qiao [23] proposed a GPS spoofing detection method based on cameras for small drones. In this study, the speed of the camera, calculated from adjacent frame images using the pyramid Lucas–Kanade (LK) optical flow method, and the speed obtained from the IMU are coupled as the reference speed of the system. The deviation between the reference speed and the instantaneous speed calculated by the GPS receiver is used for spoofing detection. The visual measurement information used in the GNSS spoofing detection is separated from the positioning system and does not fuse with the visual/GNSS information to improve the robustness of the navigation system. Furthermore, the existing coupled visual/inertial/GNSS positioning algorithm does not consider the adverse effect of GNSS spoofing. When the GNSS receiver locks the spoofing signal, the integrated navigation system is deceived and induced to the wrong position.

**Figure 1:**
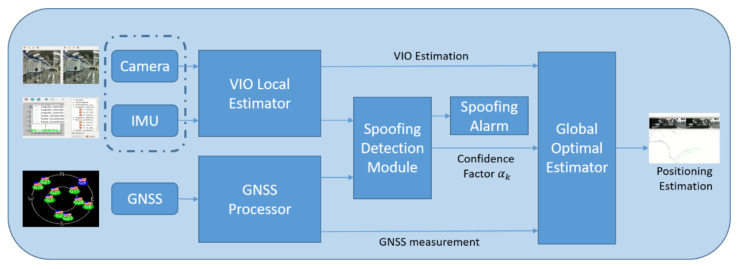
An illustration of the proposed algorithm. The spoofing detection module evaluates the VIO estimation and GNSS measurement and then transfers the confidence factor to the global optimal estimator.

In order to improve the performance of the aided spoofing detection method using supplementary measurements and protect visual/inertial/GNSS integrated systems from GNSS spoofing, a novel spoofing detection method based on a coupled visual/inertial/GNSS algorithm for UVs/UAVs is proposed in this paper. The coupled visual/inertial/GNSS positioning algorithm has been widely discussed in simultaneous localization and mapping (SLAM) and other fields. In this paper, a well-known, open-source, optimization-based coupled VIO/GNSS positioning algorithm VINS-Fusion is referred to. The KITTI dataset [24] is used to verify the vulnerability of VINS-Fusion to spoofing attacks. Then, a spoofing detection algorithm based on Chi-square detection is proposed to identify the presence of a spoofing attack, in which a test statistic is calculated based on the residuals and error covariance matrix. A GNSS confidence factor is defined to modify the coupled positioning algorithm and to prevent false spoofing alarms. To evaluate the proposed spoofing detection method, a visual/inertial/GNSS integrated system was established. The experiments were conducted both for the KITTI dataset and the real GNSS spoofing scenarios. The framework structure of the spoofing detection and coupled positioning algorithm proposed in this paper is illustrated in Figure 1.

The main contributions of this paper can be summarized as follows:

1. A novel spoofing detection method using visual/inertial estimations as supplementary measurements is proposed, with the spoofing detection factor calculated by residuals and the error covariance matrix;
2. The spoofing detection and the coupled visual/inertial/GNSS positioning estimation are both considered as part of the coupled positioning system. The vulnerability of the coupled visual/inertial/GNSS algorithms under spoofing attacks is discussed;
3. The least squares solution for GNSS measurement under spoofing interference is analyzed in order to represent the mechanism of spoofing errors. Suddenly changed and induced spoofing are discussed and modeled;
4. The experiments for the proposed spoofing detection method and the modified coupled positioning algorithm were conducted both with a dataset and real GNSS spoofing scenarios. The effectiveness of the detection and the superiority of the modified coupled positioning algorithm under GNSS spoofing attacks are shown.

The rest of the paper is organized as follows. In Section 2, the GNSS model under spoofing attack and the coupled VIO/GNSS algorithm are introduced. Then, the proposed GNSS spoofing detection method is presented in Section 3. In Section 4, the performances of the spoofing detection method and the coupled positioning algorithm are evaluated both through dataset experiments and real spoofing experiments. Section 5 concludes the paper.

## 2. GNSS Measurement and Coupled VIO/GNSS Navigation System

### 2.1. GNSS Measurement under Spoofing Attack

The GNSS receiver locks and processes positioning signals transmitted by GNSS satellites. It calculates its own position from the pseudo-range measurements and positions of multiple satellites. The pseudo-range measurement can be expressed as:

(1) $$\rho_{i}=r_{i}+c\delta t_{r}+{\tilde{\varepsilon }}_{\rho }^{i}$$

where $\rho_{i}$ is the pseudo-range measurement from the receiver to the ith satellite. $r_{i}$ is the true distance from the receiver to the ith satellite and $r_{i}=\sqrt{{\left(x-x_{i}\right)}^{2}+{\left(y-y_{i}\right)}^{2}+{\left(z-z_{i}\right)}^{2}}$. c is the speed of light. $P_{GNSS}={\left[x,y,z\right]}^{T}$ is the coordinate of the receiver and $P_{i}={\left[x_{i},y_{i},z_{i}\right]}^{T}$ is the coordinate of the ith satellite in Earth-centered, Earth-fixed (ECEF) coordinates. $\delta t_{r}$ is the clock difference of the receiver. ${\tilde{\varepsilon }}_{\rho }^{i}$ includes the satellite clock difference, ionospheric and atmospheric delay, receiving noise, multipath errors, and other kinds of errors. 

${\tilde{\varepsilon }}_{\rho }^{i}$ is generally calculated or estimated from the information in the ephemeris, so it can be considered a known quantity. Generally, GNSS spoofing attacks deceive the GNSS receiver into registering the wrong position by making the receiver process false pseudo-range measurements. In contrast with Equation (1), the pseudo-range measurement under spoofing interference can be expressed as:

(2) $$\rho_{si}=\rho_{i}+\eta_{si}=r_{i}+c\delta t_{r}+{\tilde{\varepsilon }}_{\rho }^{i}+\eta_{si},$$

where $\rho_{si}$ is the pseudo-range from the receiver to the ith satellite under spoofing and $\eta_{si}$ is the spoofing deviation added by the spoofer. Under spoofing conditions, the receiver solves the equations including spoofing errors as follows [1]:

(3) $$G\left[\begin{array}{c} \Delta x_{s}\\ \Delta y_{s}\\ \Delta z_{s}\\ \Delta \delta t_{rs} \end{array}\right]=b_{s}.$$

where $G=\left[\begin{array}{cccc} -L_{1,x}\left({\tilde{P}}_{GNSS,k-1}\right) & -L_{1,y}\left({\tilde{P}}_{GNSS,k-1}\right) & -L_{1,z}\left({\tilde{P}}_{GNSS,k-1}\right) & 1\\ -L_{2,x}\left({\tilde{P}}_{GNSS,k-1}\right) & -L_{2,y}\left({\tilde{P}}_{GNSS,k-1}\right) & -L_{2,z}\left({\tilde{P}}_{GNSS,k-1}\right) & 1\\ \cdots & \cdots & \cdots & \cdots \\ -L_{N,x}\left({\tilde{P}}_{GNSS,k-1}\right) & -L_{N,y}\left({\tilde{P}}_{GNSS,k-1}\right) & -L_{N,z}\left({\tilde{P}}_{GNSS,k-1}\right) & 1 \end{array}\right]$, $b_{s}=\left[\begin{array}{l} \rho_{1}+\eta_{s1}-r_{1}\left({\tilde{P}}_{GNSS,k-1}\right)-c\delta t_{r,k-1}-{\tilde{\varepsilon }}_{\rho }^{1}\\ \rho_{2}+\eta_{s2}-r_{2}\left({\tilde{P}}_{GNSS,k-1}\right)-c\delta t_{r,k-1}-{\tilde{\varepsilon }}_{\rho }^{2}\\ \cdots \\ \rho_{N}+\eta_{sN}-r_{N}\left({\tilde{P}}_{GNSS,k-1}\right)-c\delta t_{r,k-1}-{\tilde{\varepsilon }}_{\rho }^{N} \end{array}\right]=b+\eta$, $\eta =\left[\begin{array}{l} \eta_{s1}\\ \eta_{s2}\\ \cdots \\ \eta_{sN} \end{array}\right]$, $b=\left[\begin{array}{l} \rho_{1}-r_{1}\left({\tilde{P}}_{GNSS,k-1}\right)-c\delta t_{r,k-1}-{\tilde{\varepsilon }}_{\rho }^{1}\\ \rho_{2}-r_{2}\left({\tilde{P}}_{GNSS,k-1}\right)-c\delta t_{r,k-1}-{\tilde{\varepsilon }}_{\rho }^{2}\\ \cdots \\ \rho_{N}-r_{N}\left({\tilde{P}}_{GNSS,k-1}\right)-c\delta t_{r,k-1}-{\tilde{\varepsilon }}_{\rho }^{N} \end{array}\right]$, $-L_{i,x}\left({\tilde{P}}_{GNSS,k-1}\right)=\frac{\partial r_{i}}{\partial x}{|}_{P={\tilde{P}}_{GNSS,k-1}}$. $\left[\begin{array}{c} {\tilde{P}}_{GNSS,k-1}\\ \delta t_{r,k-1} \end{array}\right]$ is the previous estimation of the position. 

When solving the least squares solution of Equation (3), there are errors caused by spoofing included in the solution as follows:

(4) $$\left[\begin{array}{c} \Delta x_{s}\\ \Delta y_{s}\\ \Delta z_{s}\\ \Delta \delta t_{rs} \end{array}\right]={\left(G^{T}G\right)}^{-1}G^{T}\left(b+\eta \right)=\left[\begin{array}{c} \Delta x\\ \Delta y\\ \Delta z\\ \Delta \delta t_{r} \end{array}\right]+{\left(G^{T}G\right)}^{-1}G^{T}\eta .$$

The GNSS receiver uses the wrong pseudo-range measurements to calculate incorrect positions and clock errors as follow:

(5) $$\begin{array}{l} \left[\begin{array}{c} {\tilde{P}}_{GNSS,k}^{spoof}\\ \delta t_{r,k}^{spoof} \end{array}\right]=\left[\begin{array}{c} {\tilde{P}}_{GNSS,k-1}\\ \delta t_{r,k-1} \end{array}\right]+\left[\begin{array}{c} \Delta x_{s}\\ \Delta y_{s}\\ \Delta z_{s}\\ \Delta \delta t_{rs} \end{array}\right]=\left[\begin{array}{c} {\tilde{P}}_{GNSS,k-1}\\ \delta t_{r,k-1} \end{array}\right]+\left[\begin{array}{c} \Delta x\\ \Delta y\\ \Delta z\\ \Delta \delta t_{r} \end{array}\right]+{\left(G^{T}G\right)}^{-1}G^{T}\eta \\ \;\;\;\;\;\;\;=\left[\begin{array}{c} {\tilde{P}}_{GNSS,k}\\ \delta t_{r,k} \end{array}\right]+\left[\begin{array}{c} \varsigma_{P,k}\\ \varsigma_{t,k} \end{array}\right] \end{array}$$

in which $\left[\begin{array}{c} {\tilde{P}}_{GNSS,k}^{spoof}\\ \delta t_{r,k}^{spoof} \end{array}\right]$ is the optimal estimated output of the receiver under spoofing attack, $\left[\begin{array}{c} {\tilde{P}}_{GNSS,k}\\ \delta t_{r,k} \end{array}\right]$ is the estimated position and clock error when there is no spoofing error, and $\left[\begin{array}{c} \varsigma_{P,k}\\ \varsigma_{t,k} \end{array}\right]={\left(G^{T}G\right)}^{-1}G^{T}\eta$ is the spoofing deviation. ${\tilde{P}}_{GNSS,k}^{spoof}$ and ${\tilde{P}}_{GNSS,k}$ are measurements in ECEF coordinates, and the outputs of the GNSS receiver are longitude, latitude, and altitude. To construct a coupled positioning algorithm, GNSS measurements in local east-north-up (ENU) or north-east-down (NED) coordinates are generally used in order to be consistent with visual/IMU measurements. By setting the origin point of the ENU or NED coordinates, GNSS measurements in local coordinates can be calculated by multiplying a transformation matrix by ${\tilde{P}}_{GNSS,k}^{spoof}$ and ${\tilde{P}}_{GNSS,k}$ on the left as follows:

(6) $$\left[\begin{array}{c} p_{k}^{GNSS}\\ \delta t_{r,k} \end{array}\right]=\left[\begin{array}{cc} T_{w}^{l} & 1 \end{array}\right]\left[\begin{array}{c} {\tilde{P}}_{GNSS,k}\\ \delta t_{r,k} \end{array}\right]$$

(7) $$\left[\begin{array}{c} p_{k}^{spoof}\\ \delta t_{r,k}^{spoof} \end{array}\right]=\left[\begin{array}{cc} T_{w}^{l} & 1 \end{array}\right]\left[\begin{array}{c} {\tilde{P}}_{GNSS,k}^{spoof}\\ \delta t_{r,k}^{spoof} \end{array}\right]=\left[\begin{array}{cc} T_{w}^{l} & 1 \end{array}\right]\left[\begin{array}{c} {\tilde{P}}_{GNSS,k}\\ \delta t_{r,k} \end{array}\right]+\left[\begin{array}{cc} T_{w}^{l} & 1 \end{array}\right]\left[\begin{array}{c} \varsigma_{P,k}\\ \varsigma_{t,k} \end{array}\right],$$

where $T_{w}^{l}$ is the transformation matrix from ECEF coordinates to ENU or NED coordinates, $p_{k}^{GNSS}$ is the GNSS measurements in ENU or NED coordinates without spoofing, and $p_{k}^{spoof}$ is the GNSS measurements in ENU or NED coordinates including spoofing deviation. The GNSS measurements are calculated as follow:

(8) $$p_{k}^{GNSS}=T_{w}^{l}{\tilde{P}}_{GNSS,k}$$

(9) $$p_{k}^{spoof}=p_{k}^{GNSS}+\varsigma_{k}^{\prime }$$

Depending on the different types and purposes of spoofing interference, $\varsigma_{P,k}$ can be modeled as a uniformly distributed error, white Gaussian noise, non-Gaussian random noise, or other forms of noise. Considering that spoofers carry out spoofing attacks with the purpose of inducing the GNSS receiver into registering another location, it seems to be more likely that spoofing errors will be mixed in as functions of time. In this paper, $\varsigma_{P,k}$ is modeled as a function of time instead of white Gaussian noise.

For most spoofing attack scenarios, high-power spoofing signals, aligned with real signals, gradually take the place of the real signals in the GNSS receiver. When the spoofer believes that the spoofing signals have already “taken over” the GNSS receiver, the next step in the spoofing procedure is undertaken. Two different kinds of spoofing split from here. In the first, a significant deviation is immediately added into the GNSS measurement, and it looks like a step response when $\varsigma_{P,k}$ is suddenly changed from 0 to a significant value. This kind of spoofing can be called suddenly changed spoofing. The other kind of spoofing adds spoofing deviation very slowly rather than suddenly. This kind of spoofing can be called inducing spoofing. The deviation in inducing spoofing usually follows a certain definite function. Without loss of generality, it is reasonable to model the spoofing deviation $\varsigma_{P,k}$ in inducing spoofing as a linear function as:

(10) $$\varsigma_{k}^{\prime }=\kappa \left(k-k_{0}\right),$$

in which $\kappa =\frac{d\varsigma_{P,k}}{dk}=const$ represents the deviation injection rate; i.e., the injection of κ meters of deviation per second into the GNSS positioning result. $k_{0}$ represents the start moment of the inducing spoofing. 

### 2.2. VIO and Coupled VIO/GNSS Algorithm

The performance of GNSS spoofing detection based on visual/inertial supplementary measurements is highly dependent on the precision of the visual/inertial positioning solution. The visual/inertial fusion algorithm is often used in the field of simultaneous positioning and mapping (SLAM). The filtering-based method and optimization-based method are two of the most commonly used methods. The filtering-based method only uses the current measurements to estimate positioning results and poses. It does not have an awareness of the whole positioning course. The optimization-based method can utilize measurements from a long period, so it has the advantage of higher accuracy. The nonlinear optimization method was selected for this paper. Based on previous work the literature [25,26], a nonlinear optimization algorithm with a key-frame sliding window is used to form the VIO. 

In a sliding window containing n frames of images, the system state vector corresponding to the nth frame $x_{k}$ and the state vector of all n frames χ can be described as:

(11) $$\chi =\left[x_{0},x_{1},\ldots ,x_{k},\ldots ,x_{n-1},x_{c}^{b},\lambda_{0},\lambda_{1},\ldots ,\lambda_{m}\right]$$

where $x_{k}=\left[p_{b_{k}}^{w},v_{b_{k}}^{w},q_{b_{k}}^{w},b_{a},b_{g}\right]$. $p_{b_{k}}^{w}$, $v_{b_{k}}^{w}$, and $q_{b_{k}}^{w}$ are the positioning solution, velocity solution, and attitude solution obtained by the VIO solution of the kth frame. $b_{a}\;\mathrm{and}\;b_{g}$ are the biases of the accelerometer and gyroscope. $\lambda_{0},\lambda_{1},\ldots ,\lambda_{m}$ are the key points of n frames. $x_{c}^{b}$ is the external parameter from the camera to the IMU.

Acquiring solutions from the VIO is a process of bundle adjustment [25]. In every optimization process, the state vector χ is adjusted to minimize the cost function, which is made up of all measurement residuals. Then, the positioning and attitude output of the VIO are obtained as the local factor of the coupled VIO/GNSS system as follows:

(12) $${\tilde{x}}_{k}=\left[p_{k}^{l},v_{k}^{l},q_{k}^{l},{\tilde{b}}_{a},{\tilde{b}}_{g}\right].$$

Since the GNSS positioning measurement and the estimation of the VIO have already been calculated, the output of the coupled navigation system is a combination of these two kinds of measurements. In this condition, GNSS spoofing is not a concern and the navigation system cannot distinguish spoofed GNSS measurements from integral GNSS measurements. The nonlinear optimization method is also used to construct the coupled navigation algorithm. The parameter to be estimated is a set of global estimations $\chi^{\prime }=\left[{\tilde{x}}_{0}^{\prime },{\tilde{x}}_{1}^{\prime },\ldots ,{\tilde{x}}_{n}^{\prime }\right]$ in which ${\tilde{x}}_{k}=\left[{\tilde{p}}_{k}^{w},{\tilde{q}}_{k}^{w}\right]$. Measurement residuals of the local factor can be expressed as [26]:

(13) $$\begin{array}{l} z_{k}^{l}-h_{k}^{l}\left(\chi^{\prime }\right)=z_{k}^{l}-h_{k}^{l}\left({\tilde{x}}_{k-1}^{\prime },{\tilde{x}}_{k}^{\prime }\right)\\ \;\;\;\;\;=\left[\begin{array}{c} q_{k-1}^{l}{}^{-1}\left(p_{k}^{l}-p_{k-1}^{l}\right)\\ q_{k-1}^{l}{}^{-1}q_{k}^{l} \end{array}\right]⊙\left[\begin{array}{c} {\tilde{q}}_{k-1}^{w}{}^{-1}\left({\tilde{p}}_{k}^{w}-{\tilde{p}}_{k-1}^{w}\right)\\ {\tilde{q}}_{k-1}^{w}{}^{-1}{\tilde{q}}_{k}^{w} \end{array}\right] \end{array}$$

where ⊙ represents quaternion subtraction. Measurement residuals of the GNSS factor can be expressed as:

(14) $$z_{k}^{GNSS}-h_{k}^{GNSS}\left(\chi^{\prime }\right)=z_{k}^{GNSS}-h_{k}^{GNSS}\left({\tilde{x}}_{k}^{\prime }\right)=p_{k}^{GNSS}-{\tilde{p}}_{k}^{w}.$$

The cost function of the navigation algorithm can be constructed from the local factor and GNSS factor as follows [26]:

(15) $$\psi^{*}=\underset{\chi^{\prime }}{argmin}\sum_{k=0}^{n}\left(\|z_{k}^{l}-h_{k}^{l}\left(\chi^{\prime }\right){\|}_{S^{l}}^{2}+\|z_{k}^{GNSS}-h_{k}^{GNSS}\left(\chi^{\prime }\right){\|}_{S^{GNSS}}^{2}\right),$$

where $\|r{\|}_{S}^{2}=r^{T}S^{-1}r$, $S^{l}$ is the error covariance matrix of the local factor, and $S^{GNSS}$ is the error covariance matrix of the GNSS factor. 

The solution of the coupled positioning algorithm is a graph-based optimization problem. This kind of optimization is a maximum likelihood estimation (MLE) problem, which consists of joint probability distributions of poses [26]. Coupled positioning results and poses from Equation (11) are the nodes of the graph that need to be optimized. The iteration-based Google Ceres Solver [27], which uses Gaussian–Newton and Levenberg–Marquadt approaches, was used to solve this optimization problem in this study. Ceres has been proven to be an efficient and reliable optimization problem solver. By minimizing the residuals in the cost function, each position estimation ${\tilde{p}}_{k}^{w}$ can be constrained by the serials of the GNSS measurements and VIO estimations, and the estimation ${\tilde{p}}_{k}^{w}$ acquires high precision thanks to the high precision of the VIO in the short term and the relatively high precision of the GNSS in the long term without spoofing interference.

However, in spoofing interference circumstances, if the GNSS receiver locks the spoofing signals and the spoofing error $\varsigma_{s}^{\prime }$ from Equation (10) has been involved in the output $p_{k}^{spoof}$, the final estimation of the coupled visual/IMU/GNSS navigation system is gradually induced to deviate from the real position to the spoofed position. To avoid the influence of spoofing interference, a GNSS confidence factor should be added to the cost function as follows:

(16) $$\psi^{*}=\underset{\chi^{\prime }}{argmin}\sum_{k=0}^{n}\left(\|z_{k}^{l}-h_{k}^{l}\left(\chi^{\prime }\right){\|}_{S^{l}}^{2}+\alpha_{k}\|z_{k}^{GNSS}-h_{k}^{GNSS}\left(\chi^{\prime }\right){\|}_{S^{GNSS}}^{2}\right),$$

in which $\alpha_{k}$ is the GNSS confidence factor. The spoofing detection method described in Section 3 can indicate the different values of $\alpha_{k}$ in different conditions.

## 3. GNSS Spoofing Detection Method

The GNSS spoofing detection method proposed in this paper is of the same type as the supplementary measurement methods introduced in Section 1. A novel spoofing detection method based on a coupled visual/inertial/GNSS positioning algorithm is proposed, which is different from the IMU-based method and utilizes VIO estimations as supplementary measurements.

Chi-square ($\chi^{2}$) detection is a widely used system-anomaly detection method based on residual analysis. If n presents a series of independent random variables following a Gaussian distribution with a mean value of 0 (that is, independent and identically distributed in a Gaussian distribution with a mean value of 0), then the random variables formed by the sum of the squares of n random variables will follow the chi-square distribution.

After aligning the time series of the GNSS measurements and VIO estimations, the real location corresponding to the kth GNSS measurement can be expressed as $p_{k}^{w}$, which the system is unable to acquire. The final location estimation of the coupled navigation system ${\tilde{p}}_{k}^{w}$ in Section 2 is the optimal estimation of real location $p_{k}^{w}$.

The residual of the GNSS measurement and the residual of the VIO estimation can be expressed as:

(17) $$\delta_{k}^{GNSS}=p_{k}^{GNSS}-p_{k}^{w},$$

(18) $$\delta_{k}^{l}=p_{k}^{l}-p_{k}^{w}.$$

Without considering the influence of spoofing interference, every GNSS measurement can be analyzed as a variable following a Gaussian distribution with a mean value of the real location $p_{k}^{w}$. So the residual of the GNSS measurement $\delta_{k}^{GNSS}$ will follow a Gaussian distribution with a mean value of 0 and an error covariance matrix of $S^{GNSS}$. Although the VIO estimation acquires a cumulative error over time, the error in each estimation is independent and will follow a Gaussian distribution. It is reasonable to make approximations at several frames between the two successive optimizations of Equation (16) that assume that the VIO estimation follows a Gaussian distribution with a mean value of the real location $p_{k}^{w}$. In another words, the residual of the VIO estimation $\delta_{k}^{l}$, in a short period, approximately follows a Gaussian distribution with an error covariance matrix of $S^{l}$. Between the two successive optimizations of Equation (14), the calculation processes of the GNSS measurements and VIO estimations are totally independent. In this short term perspective, the distributions of $\delta_{k}^{GNSS}$ and $\delta_{k}^{l}$ are independently and identically distributed in a Gaussian distribution.

The nature of a Gaussian distribution entails that adding and subtracting the independent Gaussian distributions still results in a Gaussian distribution. The residual between the GNSS measurement and the VIO estimation is calculated as follows:

(19) $$\delta_{k}=p_{k}^{GNSS}-p_{k}^{l}=\delta_{k}^{GNSS}-\delta_{k}^{l},$$

in which $\delta_{k}$ still approximately follows a Gaussian distribution with an error covariance matrix of $S^{GNSS}+S^{l}$. 

To execute the Chi-square detection test, a Chi-square detection factor can be constructed from normalized residuals as follows:

(20) $$l_{k}=\sum_{i=k-N+1}^{k}\delta_{i}{}^{T}{\left(S^{GNSS}+S^{l}\right)}^{-1}\delta_{i},$$

in which $\delta_{k}{}^{T}{\left(S^{GNSS}+S^{l}\right)}^{-1}\delta_{k}$ is the normalized residual at the testing moment k, and $l_{k}$ is the sum of all normalized residuals in a testing window with a length of N, which means there will be N GNSS VIO residuals in the testing window up to the testing moment. Under normal circumstances, $\delta_{k}$ follows a Gaussian distribution, so $l_{k}$ is a Chi-square random variable and follows the Chi-square distribution. The value of the degrees of freedom in this Chi-square distribution is determined by the dimensions of the residual $\delta_{k}$ and the length of the testing window N.

The last procedure in Chi-square detection is hypothesis verification. Based on the degrees of freedom and the selected false detection rate Pf, a Chi-square threshold Th from the table of the Chi-square distribution can be set up to test the Chi-square factor $l_{k}$. Considering the tendency for error accumulation of VIO estimations and the non-Gaussian nature of the VIO residuals, spoofing detection must make some modifications to the Chi-square detection. The spoofing detection threshold should be considered as an amplification of the Chi-square detection threshold by multiplying by a non-Gaussian factor β (β>1). The hypothesis verification is as follows:

(21) $$hypothesis\;verification:hy=\left\{\begin{array}{c} 1:l_{k}\geq \beta Th\\ 0:l_{k}<\beta Th \end{array}\right.$$

where the result of the hypothesis verification being 1 means that $l_{k}$ likely follows a Chi-square distribution with a probability of 1−Pf, and the result of the hypothesis verification being 0 means that $l_{k}$ is likely to not follow a Chi-square distribution. 

Under spoofing circumstances, if the GNSS receiver locks the spoofing signals and spoofing deviation $\varsigma_{k}^{\prime }$ from Equation (10) has been involved in the output $p_{k}^{spoof}$, the spoofing deviation will also be involved in the residual as follows:

(22) $$\delta_{k}^{\prime }=p_{k}^{spoof}-p_{k}^{l}=p_{k}^{GNSS}+\varsigma_{s}^{\prime }-p_{k}^{l}=\delta_{k}+\varsigma_{k}^{\prime },$$

in which the residual $\delta_{k}^{\prime }$ has the form of a Gaussian distribution plus spoofing deviation. In these circumstances, the Chi-square detection factor can be described as:

(23) $$l_{k}=\delta_{k}^{\prime }=\sum_{i=k-N+1}^{k}{\left(\delta_{k}+\varsigma_{s}^{\prime }\right)}^{T}{\left(S^{GNSS}+S^{l}\right)}^{-1}\left(\delta_{k}+\varsigma_{s}^{\prime }\right)=l_{k,real}+\;\sum_{i=k-N+1}^{k}\varsigma_{s}^{\prime }{}^{T}{\left(S^{GNSS}+S^{l}\right)}^{-1}\varsigma_{s}^{\prime }+\sum_{i=k-N+1}^{k}\varsigma_{s}^{\prime }{}^{T}{\left(S^{GNSS}+S^{l}\right)}^{-1}\varsigma_{s}^{\prime }+\;\sum_{i=k-N+1}^{k}\varsigma_{s}^{\prime }{}^{T}{\left(S^{GNSS}+S^{l}\right)}^{-1}\varsigma_{s}^{\prime }=l_{k,real}+l_{spoof}$$

The Chi-square detection factor under spoofing interference in Equation (20) includes two separated parts. $l_{k,real}$ represents the real factor without the influence of spoofing, and $l_{spoof}$ is from the spoofing interference. The numerical relationship between $l_{k,real}$ and $l_{spoof}$ can be analyzed from different spoofing patterns and different laws of change for the spoofing deviation $\varsigma_{k}^{\prime }$.

The residual between the GNSS measurement and the VIO estimation $\delta_{k}$ has the magnitude of a meter and is constrained by the accuracy of the GNSS receiver and the VIO estimator. The error covariance matrix $S^{GNSS}$ and $S^{l}$ also indicate this character of $\delta_{k}$. The quantity of $l_{spoof}$ is decided by the relation between the magnitude of the spoofing deviation $\varsigma_{k}^{\prime }$ and the residual $\delta_{k}$.

In Section 2.1, suddenly changed spoofing and inducing spoofing were introduced. The spoofing deviation $\varsigma_{k}^{\prime }$ in suddenly changed spoofing interference has a significant value from the point that the deviation begins. To fulfill the purpose of spoofing the GNSS receiver into the wrong place, the deviation has to be obvious enough. The magnitude of the spoofing deviation $\varsigma_{k}^{\prime }$ can be tens or hundreds of meters, which is much larger than the residual $\delta_{k}$.

The spoofing deviation $\varsigma_{k}^{\prime }$ in inducing spoofing is much more complicated. If the spoofing deviation $\varsigma_{k}^{\prime }$ accumulates very quickly and becomes enormous during the testing window, it can be similar to suddenly changed spoofing. Only when the deviation injection rate κ in Equation (10) is small enough can the spoofing deviation $\varsigma_{k}^{\prime }$ infect the positioning result smoothly and progressively.

By providing an evaluation of GNSS quality from spoofing detection, the GNSS confidence factor $\alpha_{k}$ in Equation (16) is varied for the different results of hypothesis verification as follows:

(24) $$\alpha_{k}=\left\{\begin{array}{c} 1\;\;\;\;\;:hy=0\\ {\frac{Th}{4l_{k}^{2}}}^{2}:hy=1 \end{array}\right..$$

When the result of hypothesis verification is 0, it indicates that the GNSS data is normal and the optimal estimation of GNSS and VIO can be operated normally. However, if the result of hypothesis verification is 1, there is a high probability that the GNSS has been spoofed and the GNSS confidence factor $\alpha_{k}$ becomes ${\frac{Th}{4l_{k}^{2}}}^{2}$. The constant factor 4 in the denominator ensures that even if the $l_{k}$ is slightly higher than the threshold, $\alpha_{k}$ can reduce the weight of the GNSS factor from 1 to under 0.25 to avoid the damage of the questionable GNSS positioning results. The $\alpha_{k}$ is a quadratic inverse proportion of $l_{k}$, and therefore suddenly changed or inducing spoofing can both make rapidly reductions of $\alpha_{k}$ to 0.01. When $\alpha_{k}$ is less than 0.01, the GNSS factor drops away to lessen the calculative burden. The benefit of this form of $\alpha_{k}$, rather than dropping the GNSS positioning results directly when hy becomes 1, is that if the detection factor $l_{k}$ has become slightly higher than the threshold Th and triggered a false alarm, there is still a chance to fix it after several optimal estimations.

## 4. Evaluation of the Proposed Spoofing Detection Method and the Coupled Algorithm 

To evaluate the performances of the proposed spoofing detection method and the VIO/GNSS coupled algorithm under spoofing circumstances, both validation with an existing dataset and experiments under real spoofing scenarios were carried out. For the dataset experiment, the KITTI [24] dataset was applied, which is widely used in computer vision, visual navigation, and SLAM. The pictures in the KITTI dataset were captured by two high-resolution color and grayscale video cameras in the mid-size city of Karlsruhe, Germany, with accurate ground truth provided by a Velodyne laser scanner and a GPS positioning system. Each picture and GPS positioning result has a highly accurate timestamp. 

### 4.1. KITTI Dataset Modification

**Figure 2:**
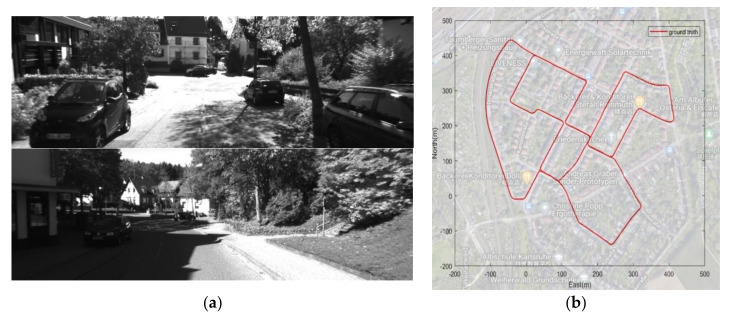
(**a**) Pictures in the KITTI dataset; (**b**) trajectory of KITTI 0_03_drive_0027 data sequence.

The KITTI 0_03_drive_0027 sequence includes 4504 pairs of pictures and the same number of GPS positioning results. Figure 2a shows the captured pictures in the dataset and Figure 2b shows the trajectory of this data sequence in Google Maps. The accuracy of the GPS positioning result in the dataset is as high as 0.05 m [24], which is high enough to be considered as ground truth. However, it is too accurate to be calculated in a coupled system. For a regular single GPS receiver, the accuracy of the GPS positioning result could be higher than 5 m, but it is harder to acquire sub-meter accuracy. Gaussian white noise with a standard deviation of 2 m was added to the GPS positioning result to simulate the real one.

**Figure 3:**
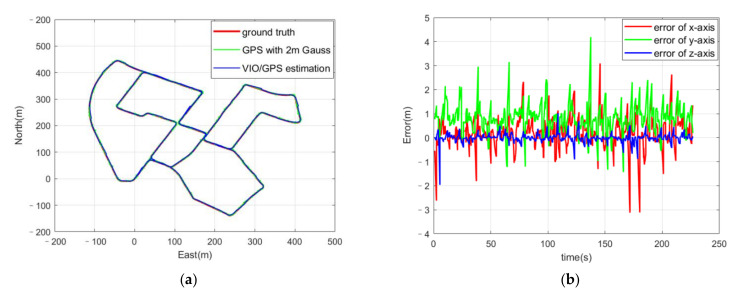
(**a**) Trajectories of ground truth, GPS positioning results with Gaussian noise, and the positioning results from the coupled GPS/VIO algorithm; (**b**) positioning errors of the three axes.

The coupled visual/IMU/GNSS positioning algorithm was operated on a Robot Operating System (ROS) on an Ubuntu 16.04 system to simulate the real-time property. Figure 3a shows the trajectory of the ground truth in red using the original high-accuracy GPS positioning results, with the trajectory of the GPS positioning results with Gaussian noise in green and the trajectory of the positioning results from the coupled VIO/GPS algorithm in black. Figure 3b shows the positioning errors from the ground truth. It indicates that if the GPS positioning results contain Gaussian noise, the positioning error of the coupled GPS/VIO algorithm is lower than the GPS positioning result. The results of the coupled GPS/VIO algorithm showed improved positioning accuracy compared to the GPS positioning results. 

**Figure 4:**
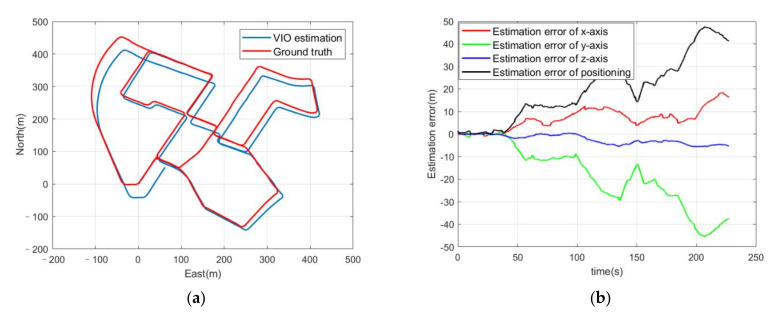
(**a**) Trajectories of ground truth and VIO estimations; (**b**) estimation errors of the three axes of the VIO and the positioning error.

To figure out the accuracy level of the VIO, an estimation experiment without GPS positioning results was carried out. The coordinate system of the VIO is a body coordinate system that does not acknowledge the relative relationship to world coordinates. Therefore, an initialization of the VIO to align it with the world coordinates must be done. The authentic GPS positioning results can be treated as the initial guesses of the positioning solutions $p_{b_{k}}^{w}$ in Equation (11), so that the optimization of Equation (15) or (16) can be convergence and the transformation matrix for the transformation from the body coordinates of the VIO to ENU or NED can be initialized. The initialization time influences the positioning result to a certain degree. If the initialization time is too short, the error in the alignment will badly increase the estimation error in the end. An initialization time of 60 s was set and proved to have good effectiveness. In the first 60 s, the positioning estimation error was constrained to less than 1 m because of the alignment. After initialization, the positioning estimation error could be considered as the error of the VIO. As shown in Figure 4b, the positioning estimation error between the VIO estimation and the ground truth was 41.34 m in the end and the average error accumulation rate was 0.105 m/s after 60s. However, the error accumulation rate of the VIO is not a fixed value. The error accumulation rate can be considered as constant for approximately every second, and the average error accumulation rate at every second can be calculated; here it was found to shift from −0.62 m/s to 0.65 m/s. The majority of the values for the error accumulation rate were under 0.4 m/s. Compared to the GPS with Gaussian noise positioning result, the VIO estimation has higher accuracy in a short period such as several seconds.

Another modification of the GPS positioning results should be undertaken to simulate the spoofed GPS positioning results. As analyzed in Section 2.1, inducing spoofing interference has an impact that gradually adds spoofing deviation to the GNSS positioning results. As shown in Equation (10), a linear model was applied to simulate the inducing spoofing deviation. The essential parameter for the inducing spoofing is the deviation injection rate α from Equation (10), as it determines the effect of inducement. If the deviation injection rate α reaches up to tens of meters per second, the residuals increase so quickly that the phenomenon may have the same characteristics as suddenly changed spoofing. The most deceptive inducing spoofing is the kind with a small deviation injection rate. It can be covered by the Gaussian noise of the GPS positioning results or the noise of VIO estimation.

To simulate this most difficult condition of inducing spoofing, the spoofed GPS positioning results were set to deviate from the real position by several meters per second and this was increased with the time. These spoofed GPS positioning results can function as a good and appropriate simulation of the GPS positioning results under strategic inducing spoofing interference.

**Figure 5:**
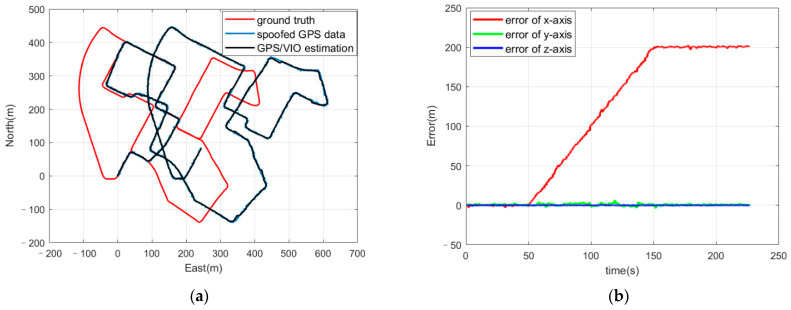
(**a**) Trajectories of ground truth, GPS positioning results with Gaussian noise and spoofing deviation, and the positioning results from the coupled GPS/VIO algorithm; (**b**) positioning errors of the coupled VIO/GPS algorithm.

The entire coupled visual/IMU/GNSS positioning algorithm was operated in ROS and fed these spoofed GPS positioning results and the corresponding visual pictures to simulate the estimation in the real world and in real time, analyzing the positioning results of the algorithm. Figure 5 shows the trajectories and the positioning errors for the positioning results and the ground truth.

The positioning results of the coupled algorithm indicated that the deviation from the spoofed GPS positioning results had a great influence on the optimal estimation. The spoofed GPS positioning results successfully induced the positioning results to the wrong place. Figure 5b shows that, when the deviation injection rate equals 1 m/s, the positioning error in the *x*-axis increases during the spoofing period and finally reaches about 200 m. As for the deviation injection rates of 2 m/s and 3 m/s, as shown in Table 1, much greater positioning errors occur during the 200 s spoofing period.

According to the positioning results with the spoofed GPS positioning results, the coupled VIO/GPS algorithm obviously becomes fatally corrupted. This is because of the unconditional trust in the GPS positioning results. The influence of the spoofing deviation is significant enough to lead the positioning estimation hundreds of meters away in several minutes. This conclusion is applicable to most of the coupled visual/IMU/GNSS algorithms in SLAM and the visual navigation field that have no robustness to inducing spoofing attacks. It is useful to deploy spoofing detection to stop the coupled visual/IMU/GNSS algorithms being deceived by inducing spoofing attacks.

### 4.2. Spoofing Detection and Coupled Positioning Algorithm Performance in Validation Using Dataset

The GNSS spoofing detection program introduced in Section 3 was added to VINS-Fusion [26] as an enhancement of this coupled GPS/VIO positioning algorithm. The GNSS spoofing detection algorithm can make up for the shortcoming of not being robust against spoofing attacks of the VINS-Fusion algorithm.

**Figure 6:**
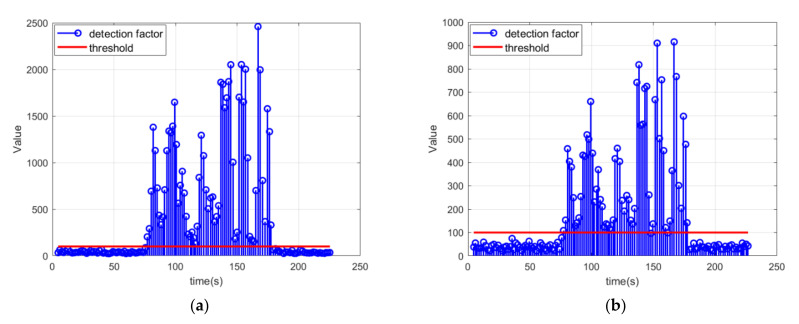
The spoofing detection results from a parallel online implementation with the coupled positioning algorithm with different deviation injection rates: (**a**) the spoofing detection results for the whole estimation with a deviation injection rate of 3 m/s; (**b**) the spoofing detection results for the whole estimation with a deviation injection rate of 2 m/s.

First, a verification of the online spoofing detection was implemented. The detection part and the optimal estimation part of the coupled positioning algorithm were run in parallel. The detection program output results individually and did not influence the estimation of the coupled positioning algorithm. The positioning estimation had undoubtedly been spoofed, just like in Figure 4a, and the results of the detection program are shown in Figure 6.

The frequency of the GPS positioning results was downsampled from 10 Hz to 2 Hz and the frequency of the visual pictures was maintained as 10 Hz to simulate the real situation. The optimal estimation of the coupled positioning algorithm was activated every 2 s to balance the computation burden and the positioning performance. Before each optimal estimation, the spoofing detection procedure was activated using the last 10 groups of VIO estimations and the GPS positioning results. However, the detection program and the positioning estimation were totally independent. This indicated that the online spoofing detection program could successfully trigger an alarm during inducing spoofing.

**Figure 7:**
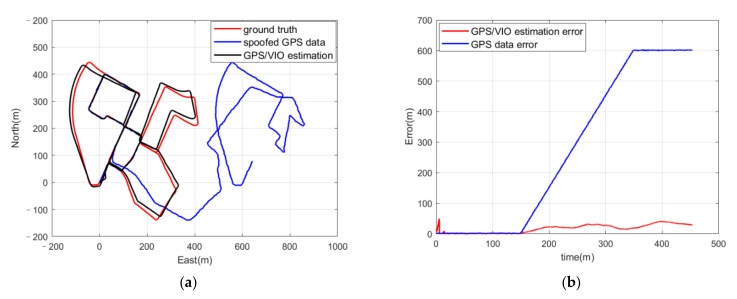
(**a**) Trajectories of ground truth in red, GPS positioning results with Gaussian noise and spoofing deviation with a deviation injection rate of 3 m/s in blue, and the positioning measurement from the coupled algorithm in black; (**b**) positioning errors of the coupled GPS/VIO algorithm in red and positioning errors of the spoofed GPS positioning results in blue.

Equation (16) can be used to execute the optimal estimation. The GNSS confidence factor $\rho_{k}$ will respond to the results of spoofing detection program. The trajectory of the coupled GPS/VIO positioning program with spoofing detection is shown in Figure 7a in black. 

Compared with the ground truth, the deceptive influence of the spoofed GPS positioning results is clear in the huge deviation of the blue line in Figure 6a. However, the coupled GPS/VIO algorithm with spoofing detection was not spoofed as in Figure 3, and the trajectory from the coupled estimation was not influenced by the spoofed GPS positioning results. Figure 7b shows the positioning errors of the spoofed GPS positioning results and the coupled estimation. It can be seen that the positioning error of the spoofed GPS positioning results grew with the inducing spoofing and reached about 600 m at 350 s, but the positioning error of the coupled GPS/VIO algorithm was constrained to under 30 m in the whole period and was not affected by the spoofed GPS positioning results thanks to the spoofing detection program.

**Figure 8:**
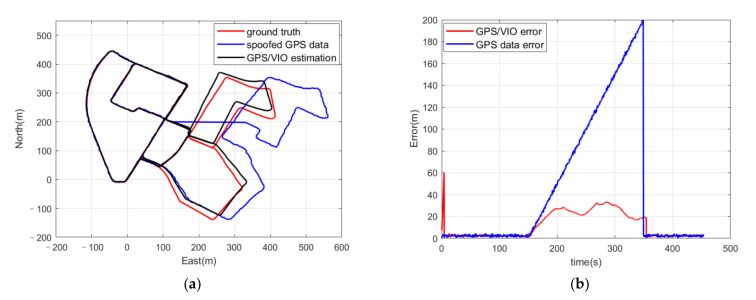
(**a**) Trajectories of ground truth in red, GPS positioning results with Gaussian noise and spoofing deviation with a deviation injection rate of 1 m/s from the 1500th frame to the 3500th frame in blue, and the positioning results from the coupled GPS/VIO algorithm in black; (**b**) positioning errors of the coupled GPS/VIO algorithm in red and positioning errors of the spoofed GPS positioning results in blue.

When the deviation injection rate of the inducing spoofing was reduced to 1 m/s, which meant that it was more deceptive and harder to detect, a similar result was obtained, as seen in Figure 8. In this case, there was no spoofing deviation in the GPS positioning results after 350 s. One hundred repetitions of the positioning estimation with these three kinds of spoofed GPS positioning results were arranged, and all of the results indicated similar conclusions for the efficiency of the inducing spoofing detection method. When the estimation was conducted with authentic GPS positioning results, represented by the first part of the spoofed GPS positioning results before 150 s, the spoofing detection program did not trigger a false alarm and the estimation results had high accuracy.

A comparison of the positioning errors of the coupled GPS/VIO algorithm with spoofing detection and the coupled GPS/VIO algorithm without detection is shown in Table 2. It can be seen that, for 200 s of inducing spoofing, the positioning error without spoofing detection was huge and, in fact, the coupled positioning algorithm was corrupted. It can be presumed that, with increases in the spoofing time, the positioning error will continually increase. The positioning error with spoofing detection was an order of magnitude smaller than without spoofing detection, and the coupled positioning algorithm could still be trusted and utilized with a slight degradation of precision.

### 4.3. GNSS Spoofing and Spoofing Detection in a Real Spoofing Experiment

**Figure 9:**
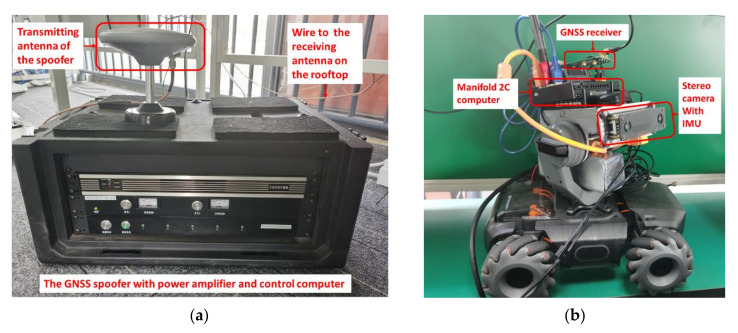
(**a**) GNSS spoofer used in the experiment; (**b**) the integrated positioning system with cameras, an IMU, and a GNSS receiver.

To evaluate the spoofing detection algorithm in real GNSS spoofing circumstances, a GNSS signal generator was used to generate spoofing signals. This kind of GNSS signal generator is specially made for experimental purposes as a spoofer. The spoofer is capable of receiving real GNSS signals and aligning the parameters of the spoofing signals to the real signals. The spoofing signals are too similar to the real ones to be distinguished by the receiver. The spoofer can transmit the spoofing signals with a SNR slightly higher than the real one. Under these conditions, some real low SNR signals that cannot be tracked by the receiver can be replaced by the similar spoofing ones. Further, the locked signals can also be replaced by the spoofing ones because of the strategy of searching for the highest correlation peak. Therefore, even if the receiver has already locked the real GNSS signal, the spoofing signal can easily take over the receiver once the spoofer has started to transmit spoofing signals. An integrated positioning system with cameras, an IMU, a GNSS receiver, and a microcomputer was set up. The real-time coupled visual/IMU/GNSS positioning algorithm and the spoofing detection algorithm were operated on this platform. The spoofer and the integrated positioning system are shown in Figure 9.

**Figure 10:**
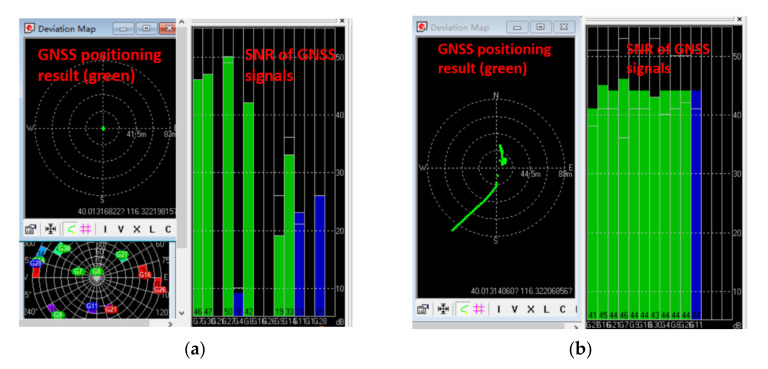
(**a**) GNSS positioning results and SNRs of the GNSS signals in normal circumstance; (**b**) GNSS positioning results and SNRs of GNSS signals under spoofing.

Figure 10 shows the GNSS positioning results and the SNR of each GNSS signal that the receiver locked in normal and in spoofing circumstances. Figure 10a shows the stationary state of the receiver and Figure 10b shows that the positioning results were deceived to move to the southwest under spoofing, although the receiver was also stationary. It can also be seen that the receiver locked more GNSS signals under spoofing, since the SNRs of the spoofing signals were generally slightly higher than the real one.

**Figure 11:**
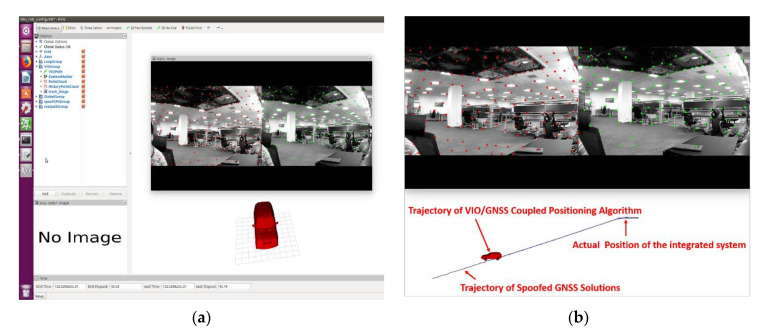
(**a**) The “rviz” interface in ROS and the stationary status of the integrated positioning system; (**b**) trajectory estimated by the coupled positioning algorithm without spoofing detection under spoofing attack.

In this experiment, the integrated positioning system remained stationary. The GNSS receiver locked real GNSS signals at the beginning with no spoofing signals. The output of the GNSS receiver initialized the coupled visual/IMU/GNSS positioning algorithm. The positioning solutions of the coupled algorithm would obviously stay close to the origin point with Gaussian noise when no spoofing signals existed. The positioning results and spoofing detection results were calculated in real-time in ROS. Figure 11a shows the “rviz” interface and the stationary status of the integrated positioning system, and the red car model represents the integrated system.

The spoofer was set to generate signals according to a certain strategy after aligning with the real signals. This meant that the positioning results calculated from the pseudo-range measurements and ephemeris from the spoofing signals would follow a certain trajectory. In this experiment, the spoofing signals were set to lead to a trajectory of uniform linear motion or uniform circular motion. To show the consequences of the spoofing attack, the spoofing detection module was not operated at first. The coupled algorithm estimates positioning solutions following Equation (15). The trajectory estimated by the coupled positioning algorithm in the real-time system under spoofing attack is shown in Figure 11b. In this spoofing attack scenario, the integrated system was obviously deceived. The positioning results showed a trajectory of uniform linear motion induced by spoofing signals.

**Figure 12:**
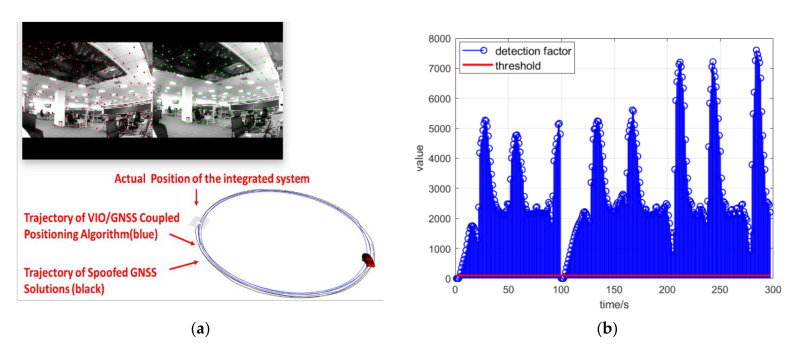
(**a**) Trajectory estimated by the coupled positioning algorithm without spoofing detection under spoofing attack; (**b**) detection statistics during the spoofing attack scenario.

**Figure 13:**
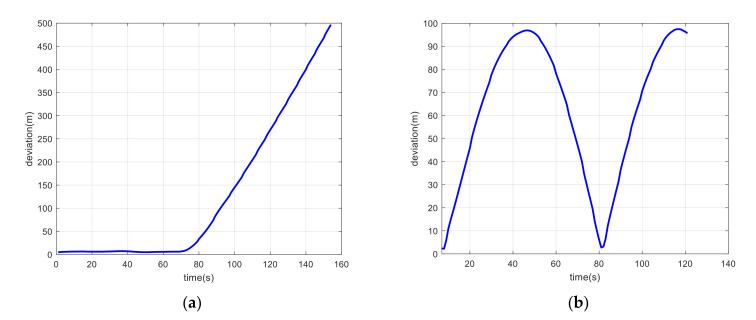
(**a**) Position deviation of the coupled positioning algorithm without spoofing detection under spoofing attack with linear motion; (**b**) position deviation of the coupled positioning algorithm without spoofing detection under spoofing attack with circular motion.

As shown in Figure 12a, the spoofing signals led to a trajectory of uniform circular motion. The positioning results of the coupled algorithm mistakenly indicated that the integrated positioning system was maneuvering and following this uniform circular motion because of spoofing signals. However, in fact, the integrated system remained stationary all the time. If the control module of the integrated system had reacted to the positioning results in this scenario, it is obvious that the integrated system would have been induced to a false position by the spoofing signals. Figure 12b shows the detection factor $l_{k}$ from Equation (20) during the spoofing attack scenario. Figure 13 shows the position deviation of the coupled positioning algorithm without spoofing detection under spoofing attack.

**Figure 14:**
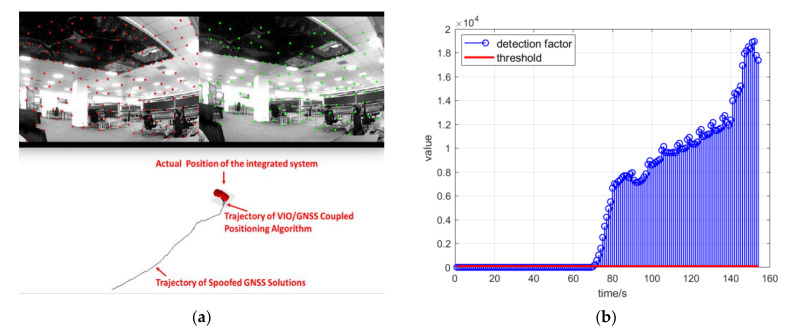
(**a**) Trajectory estimated by the coupled positioning algorithm with spoofing detection under spoofing attack; (**b**) detection statistics during the spoofing attack scenario.

As a comparison, the spoofing detection module designed with Equation (21) was initiated to execute the optimal estimation with the spoofing detection algorithm in the next experiment. As explained in Equations (21)–(24), the GNSS confidence factor $\alpha_{k}$ responds to the results of the spoofing detection algorithm. The trajectory estimated by the coupled positioning algorithm under spoofing attack is shown in Figure 14a. Figure 14b shows the detection statistics during the spoofing attack scenario.

**Figure 15:**
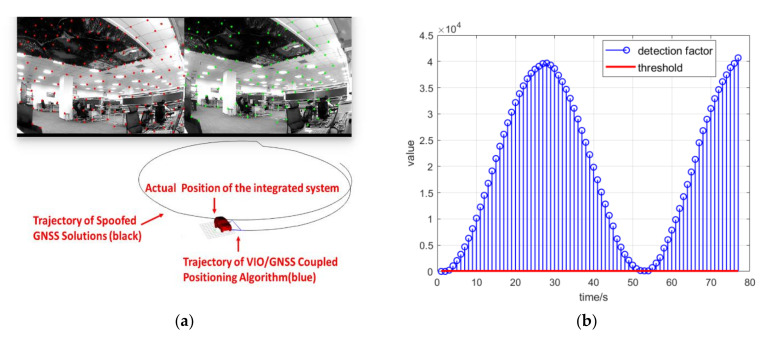
(**a**) Trajectory estimated by the coupled positioning algorithm with spoofing detection under spoofing attack; (**b**) detection statistics during the spoofing attack scenario.

When spoofing signals led to a trajectory of uniform circular motion, the spoofing position periodically returned to close to the authentic position. After the spoofing detection module started to work, the GNSS confidence factor $\alpha_{k}$ decreased rapidly to 0 because of the spoofing deviation. As shown in Figure 15a, the positioning result of the coupled algorithm could resist the influence of the spoofing attack and presented the static condition of the integrated system. Although the positioning result of the GNSS followed the circular trajectory and was deceived by the spoofing signals, the overall positioning result of the coupled algorithm remained stationary. Figure 15b shows the detection statistics during the spoofing attack scenario. When the spoofing position was close to the authentic position, the detection statistics fell back below the spoofing detection threshold. The GNSS confidence factor $\alpha_{k}$ could recover to 1 and lead the GNSS messages back to the optimal estimation. However, the GNSS confidence factor $\alpha_{k}$ would decrease to 0 again when the spoofing position drifted from the authentic position.

**Figure 16:**
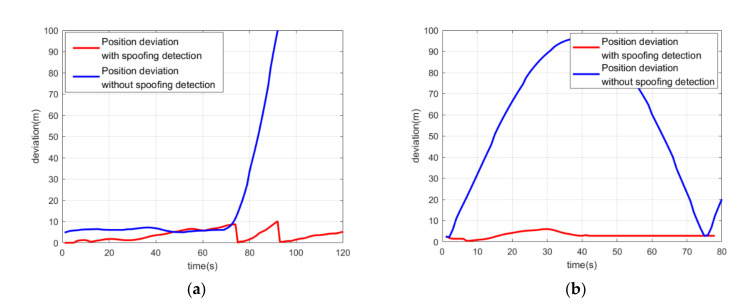
(**a**) Position deviation of the coupled positioning algorithm with spoofing detection under spoofing attack with linear motion; (**b**) position deviation of the coupled positioning algorithm with spoofing detection under spoofing attack with circular motion.

Figure 16 shows the position deviation of the coupled positioning algorithm with spoofing detection under spoofing attack. Compared with Figure 13, the results of the real GNSS spoofing experiments indicate that the spoofing detection module based on the detection algorithm is useful and efficient. It is a necessary supplement for the coupled visual/IMU/GNSS positioning algorithm against spoofing attack threats.

## 5. Conclusions

GNSS spoofing has been proven to be one of the most dangerous threats to navigation systems. If they are unaware of the existence of GNSS spoofing interference, coupled navigation systems will be corrupted by the damage caused by spoofed GNSS data. In this paper, a spoofing detection method based on a coupled visual/inertial/GNSS positioning algorithm to alert the integrated system of spoofing attack was proposed. The least squares solution of the GNSS measurement under spoofing interference was analyzed to represent the mechanism of the spoofing error. To achieve high-level positioning accuracy, an open-source, optimization-based, coupled VIO/GNSS positioning algorithm was introduced. Then, the damage caused by the spoofed GNSS measurements to the coupled positioning algorithm was analyzed. A modification through the addition of a GNSS confidence factor was made to adapt to the spoofing scenario.

The spoofing detection method proposed in this paper was based on Chi-square detection. The residuals between the GNSS measurements, VIO estimations, and real position were analyzed separately. Spoofing detection statistics were constructed from the residuals and error covariance matrixes.

The KITTI dataset was introduced and modified to simulate a spoofing scenario in this paper. The operating results of the coupled positioning algorithm with the spoofed GPS positioning results indicated that the positioning estimation had been deceived about the authentic location with a spoofed location. The experiment involving the coupled positioning algorithm with spoofing detection indicated that, even with a deviation injection rate in inducing spoofing as small as 1 m/s, the spoofing detection program worked and prevented the positioning result from being induced to a deceptive position. Similar results were found when the deviation injection rate was increased. The positioning errors were compared in two conditions, with and without spoofing detection, and it was found that the former was constrained and at least an order of magnitude smaller than the latter.

The real spoofing experiment was undertaken using a GNSS spoofer. A spoofing attack was executed after the GNSS receiver of the integrated positioning platform had already locked real GNSS signals. The positioning results of the coupled algorithm were induced to a certain deceptive position or moved following the spoofing trajectory. The spoofing detection algorithm and spoofing detection module were verified to be good supplements to the coupled positioning algorithm against spoofing attacks.

In the results from this paper, the vulnerability of the coupled visual/inertial/GNSS system under inducing spoofing interference was apparent. The spoofing detection method and the modification made to the coupled positioning algorithm proposed in this paper were able settle this problem up to a point. This system could potentially be used in any coupled navigation system that is equipped with a camera, IMU, and GNSS receiver, such as most UVs and UAVs.

## Figures and Tables

**Table 1:** Positioning errors of the coupled GPS/VIO algorithm with different spoofing deviation injection rates.

Deviation Injection Rate	Duration	Positioning Error
1 m/s	200 s	199.5 m
2 m/s	200 s	395.3 m
3 m/s	200 s	589.7 m

**Table 2:** Positioning errors of the coupled GPS/VIO algorithm with and without spoofing detection.

Deviation Injection Rate	Positioning Error with Spoofing Detection	Positioning Error without Spoofing Detection
1 m/s	19.2 m	199.5 m
2 m/s	29.5 m	395.3 m
3 m/s	29.0 m	589.7 m

## Data Availability

Not applicable.
